# Kinetics of autophagic activity in nanoparticle-exposed lung adenocarcinoma (A549) cells

**DOI:** 10.1080/27694127.2023.2186568

**Published:** 2023-03-15

**Authors:** Arnold Sipos, Kwang-Jin Kim, Constantinos Sioutas, Edward D. Crandall

**Affiliations:** a Will Rogers Institute Pulmonary Research Center and Hastings Center for Pulmonary Research, Keck School of Medicine, University of Southern California, Los Angeles, CA, USA; b Department of Pathology, Keck School of Medicine, University of Southern California, Los Angeles, CA, USA; c Department of Physiology and Neurosciences, Keck School of Medicine, University of Southern California, Los Angeles, CA, USA; d Department of Pharmacology and Pharmaceutical Sciences, School of Pharmacy, University of Southern California, Los Angeles, CA, USA; e Department of Biomedical Engineering, Viterbi School of Engineering, University of Southern California, Los Angeles, CA, USA; f Sonny Astani Department of Civil and Environmental Engineering, Viterbi School of Engineering, University of Southern California, Los Angeles, CA, USA; g Mork Family Department of Chemical Engineering and Materials Science, Viterbi School of Engineering, University of Southern California, Los Angeles, CA, USA

**Keywords:** autophagy, autophagic capacity, ambient ultrafine particles, polystyrene nanoparticles, Rapamycin

## Abstract

Autophagy, a homeostatic mechanism, is crucial in maintaining normal cellular function. Although dysregulation of autophagic processes is recognized in certain diseases, it is unknown how maintenance of cellular homeostasis might be affected by the kinetics of autophagic activity in response to various stimuli. In this study, we assessed those kinetics in lung adenocarcinoma (A549) cells in response to exposure to nanoparticles (NP) and/or Rapamycin. Since NP are known to induce autophagy, we wished to determine if this phenomenon could be a driver of the harmful effects seen in lung tissues exposed to air pollution. A549 cells were loaded with a fluorescent marker (DAPRed) that labels autophagosomes and autolysosomes. Autophagic activity was assessed based on the fluorescence intensity of DAPRed measured over the entire cell volume of live single cells using confocal laser scanning microscopy (CLSM). Autophagic activity over time was determined during exposure of A549 cells to single agents (50 nM Rapamycin; 80 μg/mL, 20 nm carboxylated polystyrene NP (PNP); or, 1 μg/mL ambient ultrafine particles (UFP) (<180 nm)), or double agents (Rapamycin + PNP or Rapamycin + UFP; concomitant and sequential), known to stimulate autophagy. Autophagic activity increased in all experimental modalities, including both single agent and double agent exposures, and reached a steady state in all cases ~2 times control from ~8 to 24 hrs, suggesting the presence of an upper limit to autophagic capacity. These results are consistent with the hypothesis that environmental stressors might exert their harmful effects, at least in part, by limiting available autophagic response to additional stimulation, thereby making nanoparticle-exposed cells more susceptible to secondary injury due to autophagic overload.

## 1. Introduction

Chronic lung diseases are associated with cellular inflammation, structural remodeling and lack or dysregulation of cell repair following repeated episodes of lung injury [1–3]. Low-grade inflammation initiated by exogenous factors (e.g., infections, cigarette smoke and/or ambient air pollution) may contribute to the pathogenesis of chronic lung diseases [3]. The initial insult and exacerbations in chronic lung disease probably are related, at least in part, to repetitive low-level cellular injury, especially in alveolar epithelial cells (AEC) [2–5].

An association between ambient air pollution and (chronic) lung diseases has long been suspected. Epidemiologic studies have found a higher prevalence of chronic lung diseases in geographical locations subject to greater air pollution [6–8]. However, the specific mechanistic links between ambient air pollution exposure and the pathobiology of chronic lung diseases have not yet been well-characterized. Importantly, the health risks of nanoparticulates (or ultrafine particles (UFP)) in ambient air have been increasingly recognized in recent years [9–11]. This fraction of inhaled particulates has higher surface area, is deposited deeper into the lung (including the alveolar air spaces) and may have greater interactions with cellular structures compared to larger particulates [12]. The cellular responses of exposure to nanoparticles (NP) are known to be dependent on their chemical (e.g., composition and surface charge) and physical (e.g., size and shape) characteristics [13–16]. Due to heterogeneity of these physicochemical properties of NP, it is difficult to generalize their effects in cells, although NP exposure has been shown to induce stress responses (e.g., mitochondrial and endoplasmic reticulum (ER) dysfunctions) in various cells and tissues [17–20].

Activation of autophagy is often observed in NP-exposed cells [21–23]. The process of autophagy was noted first as a mechanism for cell survival during periods of starvation [24]. Autophagy has been known to be primarily a defense mechanism important in maintaining cellular homeostasis, regulation of which appears to be complex [25,26]. Autophagic activity (or flux) can be triggered by many cellular insults and contributes to numerous downstream cellular effects [27]. However, it is unclear if there is a limit to autophagic activity (or autophagic capacity) beyond which cellular defenses may be overwhelmed and cell death mechanisms can be activated. It is known that when cells undergo severe starvation and/or experience excess oxidant stress, autophagic processes may be insufficient to overcome the challenge and cell death (e.g., apoptosis) ensues [28,29]. Autophagy has a dual role in disease processes; for example, in certain cancers, it can promote adaptation and survival of tumor cells, as opposed to autophagy-deficient cancers in which stimulation of autophagic activity can contribute to the elimination of tumor cells [30,31]. Numerous studies are focusing on the dual role of autophagy in disease processes and the development of autophagy modulators that can be utilized for targeted therapy. Nanomaterials in general modulate autophagic processes and may present novel approaches to augment traditional therapeutic strategies [32]. Autophagy modulation can affect tumorigenesis, metastasis and drug resistance against anticancer agents [33,34].

As in all organs, autophagy plays a crucial role in lung development and homeostasis [35–37]. Unlike other organs, however, the lung is also challenged directly by environmental stressors (e.g., airborne pathogens and pollutants), as a result of which autophagy is especially important for the maintenance of normal lung function. For example, reduced autophagy caused hypersensitivity to oxidative stress and increased inflammation in asthma [38]. Similarly, blockade of autophagy may lead to pulmonary fibrosis following silica NP exposure [39]. It seems that reduction in baseline autophagy is deleterious, although overstimulation of (selective) autophagy can also produce catastrophic cellular events [40]. Involvement of autophagy in lung diseases has been recognized [41], but the question of how much autophagic capacity is needed to maintain normal cellular homeostasis when challenged requires further investigation.

We have previously reported that AEC internalize polystyrene nanoparticles (PNP) and can exhibit resultant mitochondrial and lysosomal dysfunction [42,43]. Exposure to UFP also activated autophagy in AEC [43]. In this study, we quantified the effects on autophagic activity in A549 cells of exposure to PNP, UFP and Rapamycin alone and to simultaneous and sequential exposure to PNP or UFP and Rapamycin.

## 2. Results

Autophagic flux at a given time point under each experimental condition was determined by live cell imaging as the fluorescence intensity of DAPRed measured over the entire volume of exposed single A549 cells in the presence of chloroquine (added to bathing fluids 1 hr prior to each time of measurement) minus that in the absence of chloroquine, all corrected for control autophagic flux. Autophagic flux under control conditions (i.e., vehicle exposure only) at each time point was determined similarly in the presence of chloroquine (added to bathing fluids 1 hr prior to each time of measurement) minus that in the absence of chloroquine (see Methods). Control autophagic flux at each time point was relatively unchanged from 0 to 24 hrs in A549 cells (see Supplementary Figure S3).

**Figure 1.**
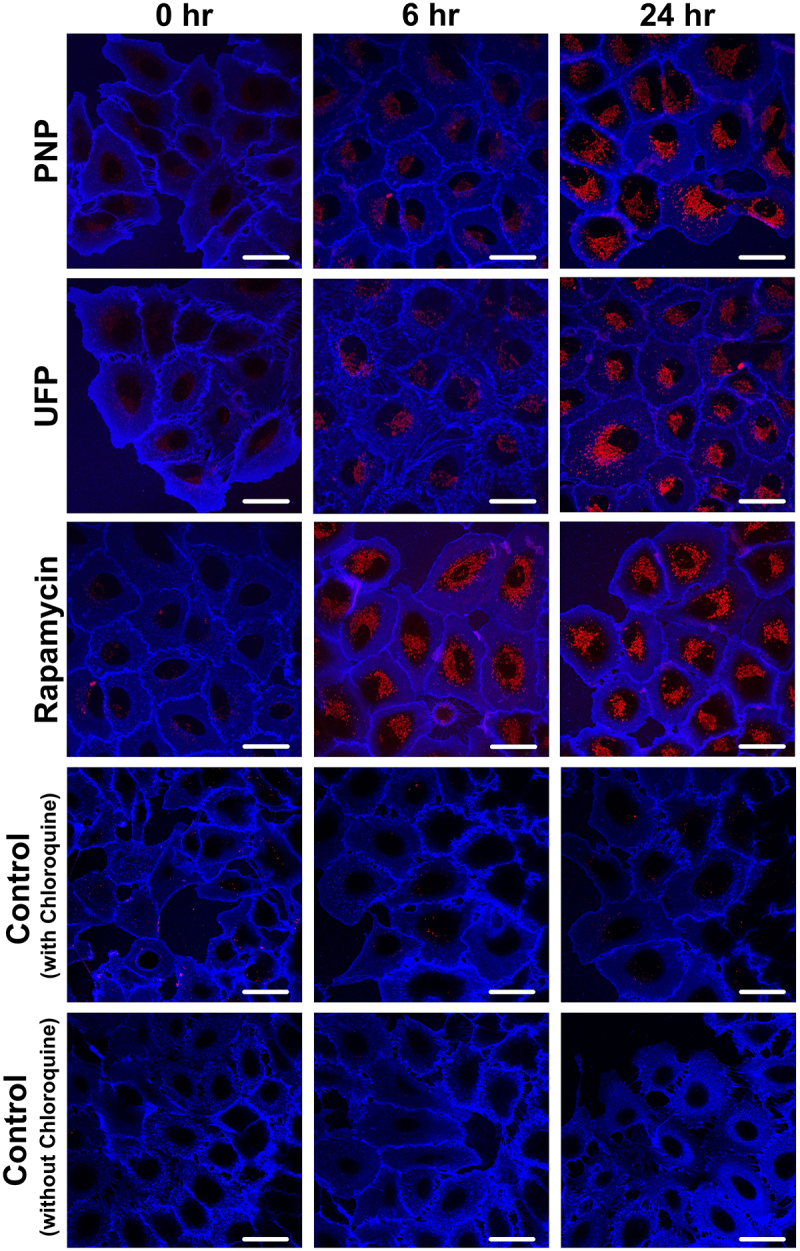
**Time-dependent activation of autophagy in A549 cells exposed apically to PNP (80 μg/mL), UFP (1 μg/mL) or Rapamycin (50 nM) as a single agent at t = 0**. Autophagic activity increased over time as shown by increased fluorescence intensity of DAPRed (red). Data were collected at each time point after 1 hr incubation with chloroquine (40 μM). Plasma membranes of A549 cells were labeled by Dylight 405-conjugated tomato lectin (blue). Scale bars are 25 μm.

Representative images of DAPRed-positive autophagosomes and autolysosomes in A549 cells are shown in Figure 1. When A549 cells were exposed apically to PNP, UFP or Rapamycin as a single agent from t = 0 to 24 hrs, minimal DAPRed fluorescence (in red) was noted at t = 0, followed by increased DAPRed fluorescence in intracellular vesicles over time. Plasma membranes of A549 cells are labeled by Dylight 405-conjugated tomato lectin (in blue).

**Figure:**
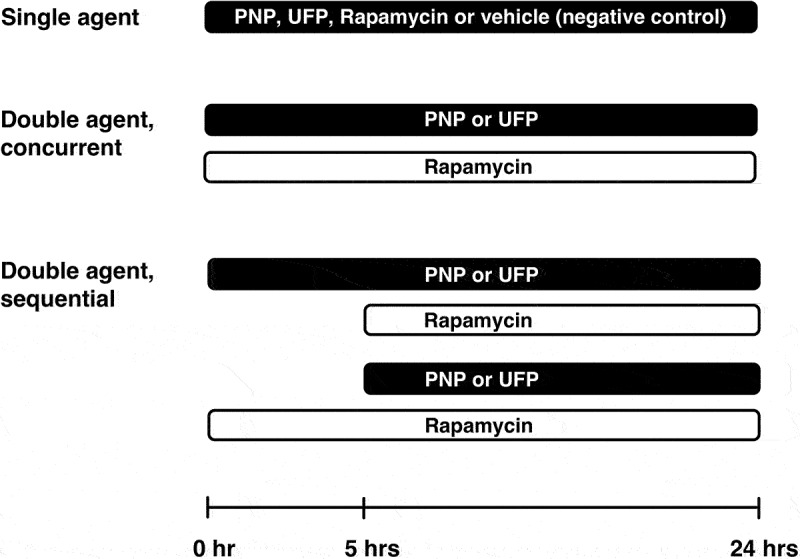

**Figure 2.**
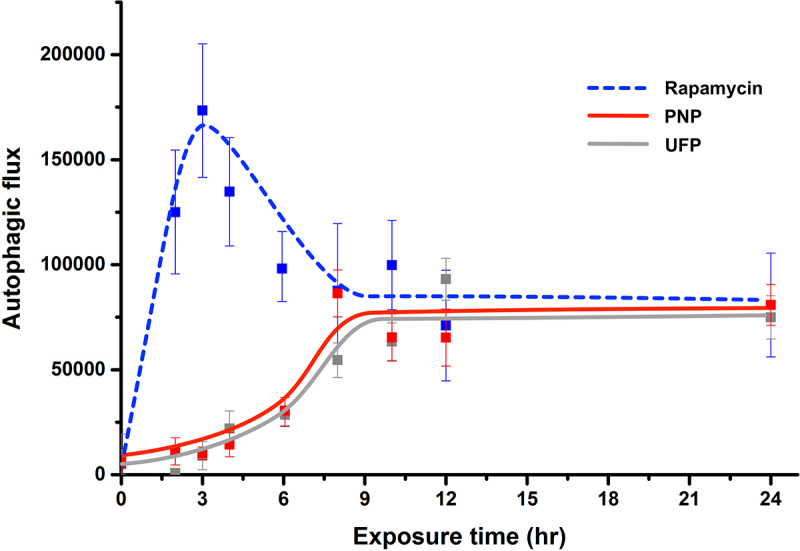
**Kinetics of autophagy activation in A549 cells exposed to a single agent using Rapamycin, PNP or UFP at t = 0 and monitored for up to 24 hrs**. Apical exposure of A549 cells to PNP (80 μg/mL, red line) or UFP (1 μg/mL, grey line) led to time-dependent gradual increases in autophagic flux. When exposed to either PNP or UFP alone at t = 0, increased autophagic flux was detected at ~3-6 hrs post exposure, reaching a peak at ~8-10 hrs. Exposure to Rapamycin (50 nM, blue line) alone at t = 0 led to a rapid increase in autophagic flux, peaking at ~3 hrs post exposure. Data at each time point were collected from 26-69 single cells. Detailed data with statistical analyses are shown in Table 1.

Composite kinetic profiles in A549 cells of autophagic flux (shown as arbitrary units (AU) of DAPRed fluorescence intensity) in response to single agent exposure at t = 0, followed by monitoring for up to 24 hrs, are shown in Figure 2. As defined in Methods, autophagic flux presented below for each experimental condition is corrected for time-matched control autophagic flux. Apical exposure of A549 cells to PNP at 80 μg/mL or UFP at 1 μg/mL led to gradual increases in autophagic flux, peaking at ~75,000 AU at ~8-10 hrs post exposure. Autophagic flux in PNP- or UFP-exposed cells remained elevated at that level from ~10 to 24 hrs. Apical exposure of A549 cells to Rapamycin led to a more rapid increase in autophagic flux, with a peak at ~175,000 AU at ~3 hrs post exposure, followed by decreasing autophagic flux to a steady state similar to that for PNP or UFP. Similar data were obtained when autophagic flux was determined using RFP-GFP-LC3B (Supplementary Figure S2).

Table 1 lists data on autophagic flux observed under nine different experimental conditions as a function of exposure time. Autophagic flux observed with an experimental condition where apical exposure of A549 cells to PNP, UFP or Rapamycin alone is listed, corresponding to the kinetic profiles shown in Figure 2. As can be seen, PNP, UFP and Rapamycin exposures all significantly increased autophagic flux as shown by increased intracellular fluorescence of DAPRed over time. Elevations in autophagic flux compared to t = 0 were first detected at 4 and 6 hrs post exposure for UFP alone and PNP alone, respectively. Autophagic flux remained significantly elevated for up to 24 hrs. Rapamycin alone induced autophagic flux as expected, detected first at 2 hrs post exposure, which is earlier than that observed for PNP or UFP exposure alone. Autophagic flux during Rapamycin exposure peaked at ~3 hrs, after which it partially decreased before reaching a steady state similar to those attained after exposure to PNP or UFP. Rapamycin exposure-induced autophagic flux was higher than PNP- or UFP-induced autophagic flux between 2 and 4 hrs of exposure, but there was no difference among exposed groups from 8 to 24 hrs of exposure.

**Table 1.** **Kinetics of autophagy activation in A549 cells after NP and/or Rapamycin exposure**. Significant differences among means at a given time point were determined by one-way ANOVA. *: p<0.05 compared to t = 0; **: p<0.01 compared to t = 0; #: p<0.05 compared to PNP exposure alone at given time; $: p<0.05 compared to UFP exposure alone at given time; γ: p<0.05 compared to PNP + Rapamycin (sequential) at given time; δ: p<0.05 compared to UFP + Rapamycin (sequential) and ε: p<0.01 compared to Rapamycin + UFP (concurrent) at given time. There were no significant differences between the two experiments for any condition at any time point.

	Exposure time (hr):	0	2	3	4	5	6	8	10	12	24
Experiment #1	PNP	5858±2964 (20)	13212±7520 (35)	10785±2970 (11)	17082±3756 (40) *		255163±20814 (35) **	88774±10290 (47) **	59309±8536 (28) **	63572±14937 (35) **	78382±8430 (41) **
	UFP	8046±933 (28)	1082±585 (27)	7243±2652 (19)	19393±6702 (21) *		246397±17262 (17) **	52778±8646 (18) **	64920±7878 (21) **	91762±11728 (22) **	75196±9703 (18) **
	Rapamycin	7763±4499 (17)	123705±50690 (28) **#$	168002±74029 (23) **$#	126304±75427 (24) **#$		98908±22451 (17) **	77352±34630 (14) **	94372±58023 (18) **	77375±21216 (21) **	80968±20965 (11) **
	Rapamycin + PNP (concurrent)	10713±2727 (19)	44324±10892 (24) ** ε	62792±12989 (26) ** ε	75496±7207 (28) ** ε		78662±18415 (17) ** ε	72878±8092 (23) **	91443±13775 (19) **	54948±13634 (16) **	67840±16691 (15) **
	Rapamycin + UFP (concurrent)	7440±3774 (16)	10702±5657 (21)	8308±7094 (23)	35425±8530 (28) **		44489±9302 (16) **	91431±16911 (21) **	74235±10333 (12) **	58702±12801 (21) **	82639±8056 (21) **
	PNP + Rapamycin (sequential)	14482±4600 (12)		12700±7746 (15)		20406±13353 (14)			99076±36173 (17) **	107142±31031 (16) **	85781±21964 (12) **
	Rapamycin + PNP (sequential)	7251±1467 (14)		116483±13089 (14) ** γ		83454±18958 (15) ** γ			79896±16076 (13) **	83634±17373 (16) **	68132±23012 (13) **
	UFP + Rapamycin (sequential)	10003±5731 (14)		34919±22520 (12) **		50157±7720 (12) **			56532±18132 (13) **	115704±16176 (11) **	68132±23012 (13) **
	Rapamycin + UFP (sequential)	8626±3787 (13)		137111±16502 (16) ** δ		100089±13856 (16) ** δ			65330±20079 (17) **	76627±18714 (15) **	99547±10779 (16) **
Experiment #2	PNP	6040±3013 (15)	8544±7419 (11)	10640±4801 (15)	10504±4508 (22) *		251245±19356 (30) **	81373±4062 (18) **	74338±10651 (21) **	68409±10010 (30) **	83089±10271 (28) **
	UFP	13478±8369 (13)	13637±288 (18)	11653±8434 (14)	23867±11174 (26) *		236241±18537 (18) **	56245±11999 (14) **	61376±8097 (17) **	95199±9367 (25) **	74448±15849 (17) **
	Rapamycin	5372±2107 (13)	128397±30773 (22) **#$	180435±90382 (17) **$#	142568±13242 (22) **#$		93350±24927 (18) **	98382±25976 (12) **	106379±25005 (14) **	63524±8763 (14) **	80434±28171 (14) **
	Rapamycin + PNP (concurrent)	11000±4272 (26)	46800±9628 (19) **ε	54696±15520 (17) **ε	73306±12467 (21) **ε		72032±6870 (16) **ε	60570±15619 (19) **	82087±20789 (23) **	62319±8426 (18) **	64392±9342 (19) **
	Rapamycin + UFP (concurrent)	7570±592 (14)	10142±4575 (15)	8555±7068 (15)	39292±9284 (13) **		45193±8335 (19) **	93140±16230 (18) **	64444±15942 (18) **	59775±12116 (18) **	87439±6459 (23) **
	PNP + Rapamycin (sequential)	9031±3727 (14)		12915±77371046 (14)		25373±7749 (15)			83486±25493 (14) **	104553±39874 (17) **	77125±22851 (15) **
	Rapamycin + PNP (sequential)	6654±1676 (15)		114657±13614 (14) **γ		94436±15372 (12) **γ			79951±8024 (14) **	86742±15021 (15) **	73662±24652 (15) **
	UFP + Rapamycin (sequential)	8544±7419 (11)		34131±15889 (11) **		50891±21285 (12) **			60798±17854 (14) **	119249±13079 (14) **	50764±23263 (15) **
	Rapamycin + UFP (sequential)	10159±4148 (14)		135533±21609 (16) **δ		102068±17578 (15) **δ			76767±14113 (15) **	86335±14539 (15) **	96300±15806 (15) **

**Figure 3.**
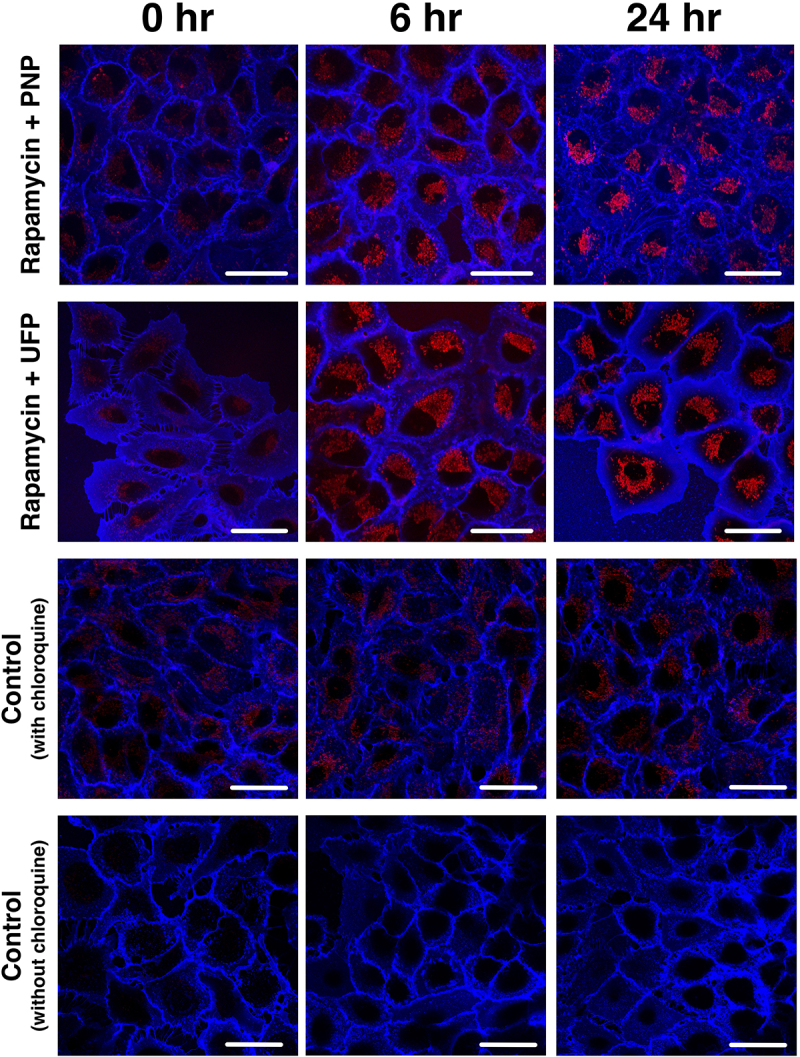
**Time-dependent activation of autophagy in A549 cells during concurrent apical exposure (at t = 0) to 50 nM Rapamycin and 80 μg/mL PNP or 1 μg/mL UFP**. Autophagic activity increased over time as seen by fluorescence intensity of DAPRed (red). Data were collected at each time point after 1 hr incubation with chloroquine (40 μM). Plasma membranes of A549 cells were labeled by Dylight 405-conjugated tomato lectin (blue). Scale bars are 25 μm.

In order to explore the effects of simultaneous exposure to NP and Rapamycin on the kinetics of autophagic flux, we next investigated autophagic flux in A549 cells exposed (at t = 0) apically to PNP at 80 μg/mL or UFP at 1 μg/mL in the concurrent presence of Rapamycin. Representative images are shown in Figure 3, in which A549 cells were exposed apically to both 50 nM Rapamycin and 80 μg/mL PNP or 1 μg/mL UFP at t = 0, demonstrating that DAPRed fluorescence activity was increased at 6 hrs and 24 hrs post-exposure.

**Figure 4.**
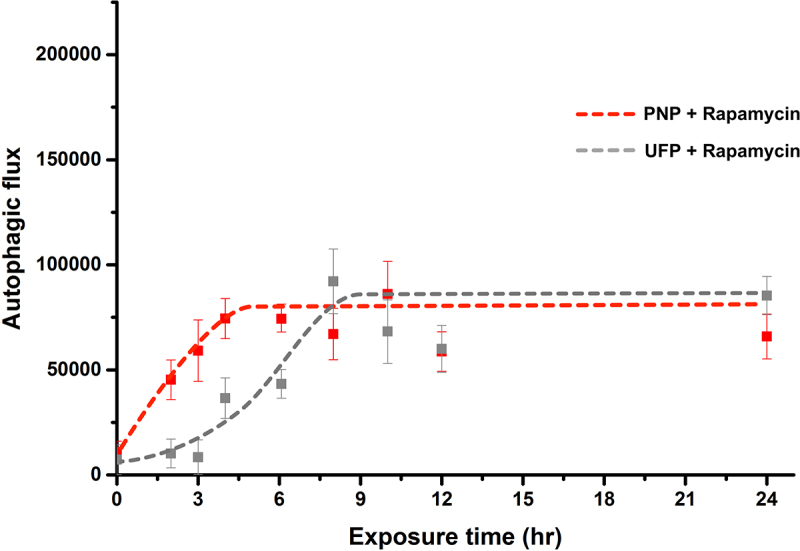
**Kinetics of autophagy activation in A549 cells concurrently exposed apically to Rapamycin and PNP or UFP at t = 0**. Concurrent apical exposure at t = 0 of A549 cells to Rapamycin (50 nM) + PNP (80 μg/mL) or Rapamycin (50 nM) + UFP (1 μg/mL) resulted in more rapid activation of autophagy in comparison to exposure to PNP or UFP alone (Figure 2). Data at each time point are from 31-49 single cells. Detailed data with statistical analyses are shown in Table 1.

Figure 4 shows the kinetic profiles of autophagic flux corresponding to the images in Figure 3. As seen, autophagic flux rapidly increased in both concurrent double exposure models of Rapamycin + PNP and Rapamycin + UFP. Maximal autophagic flux in both double exposure experiments was similar to that at steady state for PNP, UFP and Rapamycin in the single exposure experiments (Figure 2). However, early autophagic flux in the concurrent double exposure experiments did not reach the level observed with single exposure to Rapamycin alone as shown in Figure 2. Similar data were obtained when autophagic flux was determined using RFP-GFP-LC3B (Supplementary Figure S2).

In Table 1, data (corresponding to those shown in Figure 4) for kinetics of autophagy activation in A549 cells concurrently exposed to Rapamycin and PNP or UFP at t = 0 are listed. Significantly elevated autophagic flux compared to t = 0 (first seen at 2 hrs post-exposure to Rapamycin alone) was seen at 2 hrs for Rapamycin + PNP and at 4 hrs for Rapamycin + UFP, and remained significantly elevated for up to 24 hrs. Autophagic flux after Rapamycin exposure alone was significantly higher than for Rapamycin + PNP-induced autophagic flux at 2 hrs, 3 hrs and 4 hrs post-exposure. Rapamycin exposure alone induced much higher autophagic flux than exposure to Rapamycin + UFP at 2 hrs, 3 hrs and 4 hrs post-exposure.

**Figure 5.**
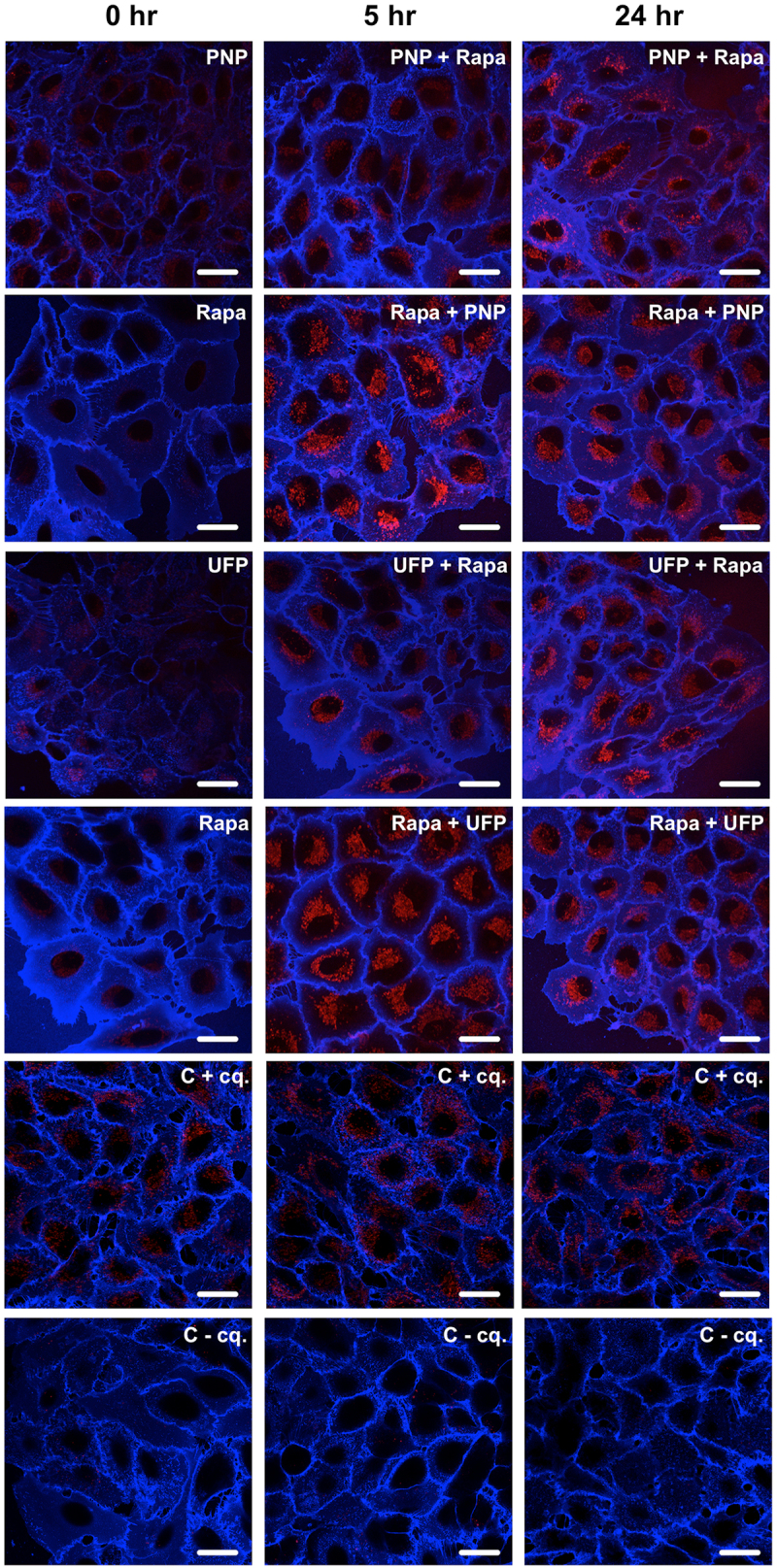
**Representative images showing the time-dependent activation of autophagy in sequential exposure experiments**. A549 cells exposed apically to a single agent (PNP, UFP or Rapamycin (Rapa)) for the first 5 hrs of the experiment, followed by exposure to the single agent plus a second agent (Rapamycin followed by PNP or UFP and PNP or UFP followed by Rapamycin) from 5 to 24 hrs. Autophagic activity increased in response to the different exposures over time as seen by fluorescence intensity of DAPRed. Data were collected at each time point after 1 hr incubation with chloroquine (40 μM). Plasma membranes of A549 cells were labeled by Dylight 405-conjugated tomato lectin (blue). C = control, cq = chloroquine. Scale bars are 25 μm.

**Figure 6.**
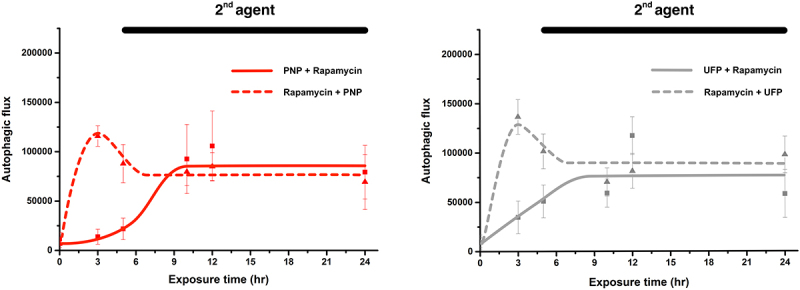
**Kinetics of autophagic activation in A549 cells exposed to two agents sequentially (one from 0 to 24 hrs and the other from 5 to 24 hrs)**. Autophagic flux was assessed in A549 cells first by exposure to PNP (solid line in left panel) or UFP (solid line in right panel) at t = 0, followed by the addition of Rapamycin at t = 5 hrs. In a different set of experiments, A549 cells were exposed to Rapamycin at t = 0, followed by the addition of PNP (dotted line in left panel) or UFP (dotted line in right panel) at 5 hrs. The overall kinetics and steady state values of autophagic flux in these reverse order sequential double exposure models were not different from those measured in single (Figure 2) or concurrent double agent exposures (Figure 4), suggesting that autophagic capacity in A549 cells is limited. Data at each time point are from 24-33 single cells. Detailed data with statistical analyses are shown in Table 1.

In order to better understand the autophagic responses to double exposures, we further assessed kinetics of autophagic activity by delaying exposure to one of the two agents in double exposure experiments. Specifically, only one agent (PNP or UFP, or Rapamycin) was present during the first 5 hrs, followed by exposure to a second agent (Rapamycin followed by PNP or UFP and PNP or UFP followed by Rapamycin) from t = 5 – 24 hrs. Representative images of these sequential exposure experiments are shown in Figure 5. As can be seen in Figure 6, when Rapamycin was used as the first agent, a rapid rise in autophagic flux was followed by decreased but elevated autophagic flux, whereas either PNP (left panel, solid line) or UFP (right panel, solid line) as the first agent resulted in autophagic flux whose magnitude remained lower in the first 5 hrs of exposure than that observed with Rapamycin alone (dotted line in both right and left panels in Figure 6). The second agent was added after 5 hrs of exposure to the first agent, after which both agents remained present for up to 24 hrs. Autophagic flux remained steady in all sequential exposure experiments, indicating that pre-exposing A549 cells to a single agent (PNP, UFP or Rapamycin) for 5 hrs did not lead to a further increase in autophagic flux in response to exposure to a different second agent from 5 hrs onward.

Figure 6 shows the kinetic profiles of autophagic flux corresponding to the images in Figure 5. Maximal autophagic flux in all sequential double exposure experiments was similar to that at steady state for PNP, UFP and Rapamycin in the single exposure experiments (Figure 2). However, early autophagic flux in the sequential double exposure experiments in which cells were first exposed to PNP or UFP did not reach the level observed after single exposure to Rapamycin alone as shown in Figure 2.

In Table 1, data are shown for kinetics of autophagic activation in A549 cells exposed to two agents sequentially (one at t = 0 and the other at t = 5 hrs). For PNP + Rapamycin, significantly elevated autophagic flux was not seen until 10 hrs post-exposure. Autophagic flux was significantly elevated in the three other experimental groups at ~3 hrs post exposure. Autophagic flux remained significantly elevated for all sequential exposure experimental groups for up to 24 hrs. For Rapamycin + PNP sequential exposure, significantly higher autophagic flux than PNP + Rapamycin sequential exposure was seen at 3 hrs and 5 hrs. For Rapamycin + UFP sequential exposure, significantly higher autophagic flux than UFP + Rapamycin sequential exposure was found at 3 hrs and 5 hrs.

## 3. Discussion

In this study, we used CLSM to estimate kinetics of time-dependent activation of autophagy in lung adenocarcinoma A549 cells. Exposure to Rapamycin alone induced a rapid rise in autophagic flux, peaking at ~3 hrs post exposure, followed by lower steady state autophagic flux for up to 24 hrs. Exposure to PNP or UFP alone also increased autophagic flux, gradually reaching steady state at ~10-12 hrs, which was maintained thereafter for up to 24 hrs.

When A549 cells were exposed concurrently to Rapamycin with PNP or UFP at t = 0, the initial rapid rise in autophagic flux disappeared. Surprisingly, the steady state level of autophagic flux remained comparable to the level observed during single exposures to Rapamycin, PNP or UFP for up to 24 hrs. Furthermore, during sequential exposure conditions, the steady state level of autophagic flux again did not exceed the steady state level observed in the case of single or concurrent exposures.

We used the small fluorescent molecule DAPRed for specific labeling of autophagosomes and autolysosomes to study autophagic activity [39]. DAPRed enables real-time assessment of autophagic activity based on its unquenched fluorescence intensity during autophagic processes, thereby being more effective for continuous quantitative assessment of autophagic kinetics than other approaches involving, for example, LC3 Western blotting or LC3 immunolabeling [44,45]. Using DAPRed with live cell imaging, it was possible to study autophagic kinetics at the single cell level. However, to ensure that data using DAPRed and LC3 are similar, we determined the degree of colocalization of DAPRed with LC3B-GFP in live A549 cells (Supplementary Figure S1). Almost complete colocalization of DAPRed-positive vesicles with LC3B-GFP-positive vesicles (~91%) was found over the entire volume of single A549 cells. Similar data on DAPRed and LC3B-GFP colocalization were recently presented by Chen and co-workers [46]. For additional verification of DAPRed results, we repeated some experiments using RFP-GFP-LC3B (Supplementary Figure S2), which showed very similar results to those using DAPRed.

Autophagy functions at a baseline level during sustained homeostasis [47], as confirmed by our measurements of control autophagic flux (Supplementary Figure S3). However, upon exposure to Rapamycin, an initial rapid elevation in autophagic flux, followed by reduction to the steady state level, was noted. Rapamycin is a small, lipophilic molecule that gains access to the cytosol considerably faster than PNP or UFP. Rapamycin is a potent inducer of autophagy via inhibition of mTOR (mammalian target of Rapamycin) [48,49], which may explain its ability to induce an early peak in autophagic flux (at ~3 hrs) as opposed to the gradual increase in autophagic flux, reaching steady state (at ~8-10 hrs), that was observed for PNP or UFP. The difference in autophagic activity between Rapamycin and PNP or UFP exposures experienced during the early phase of autophagic activation (0-9 hrs post exposure) might also suggest that Rapamycin and PNP or UFP exert their effects on autophagy via different pathways. The Rapamycin-induced early peak in autophagic flux was eliminated by concurrent exposure of Rapamycin with PNP or UFP (Figure 4). This suggests an inhibitory interaction of PNP or UFP with Rapamycin (e.g., interaction with the protein corona of NP might make Rapamycin less able to inhibit mTOR). Further experimental explorations will be needed to help clarify these phenomena.

Elevation in autophagic flux required more time after exposure to PNP or UFP compared to Rapamycin exposure, consistent with our prior findings that PNP entry into primary rat AEC was found to take several hours [42]. Delayed cellular entry of NP would explain the delayed increase in autophagic activity observed after exposure to PNP and UFP.

Once autophagic activity was induced, it reached a steady state over time which was about the same level regardless of exposure conditions (i.e., to Rapamycin, PNP, UFP and combinations thereof). Furthermore, when exposures were combined in a concurrent or sequential fashion, steady state autophagic flux did not increase beyond the steady state observed for single exposures, suggesting a possible limit to maximal autophagic capacity under these experimental conditions. This unexpected finding might be attributable, at least in part, to the availability of lipid membranes for autophagosome formation. Double membranes of autophagosomes are derived primarily from ER, which has a capacity limit in a given cell [50,51]. Similarly, the capacity of lysosomal degradation is also expected to have a maximum, although number and size of lysosomes can increase but not exceed a limit [52].

In prior work, we reported that PNP-exposed AEC accumulate NP in intracellular vesicles (autophagosomes and lysosomes) [42]. Intracellular PNP content reached steady state and increasing exposure concentration did not further elevate it, although intracellular accumulation of PNP became more rapid [42]. Because the intracellular presence of PNP induced autophagy (and only a fraction of total intracellular PNP content remained free in the cytosol) in AEC, the ceiling of intracellular PNP content may be related to a capacity limitation of autophagic activity. However, delayed uptake of the agents that stimulate autophagy cannot explain the observed maximum levels in autophagic activity since autophagic flux at 48 hrs of exposure is not different from autophagic flux at 24 hrs of exposure (Supplementary Figure S4).

Similar results on kinetics of autophagic activity were reported when primary motor neurons were subjected to oxygen and glucose deprivation [53]. In this case, autophagic activity was induced after ~2 hrs and reached its maximum after 5 hrs. In addition, when autophagic activity was assessed in an in vivo mouse model of sepsis, peak autophagic activity was observed at 6 hrs in liver, which returned to baseline by 24 hrs post exposure [54]. This result also confirmed that data on kinetics and capacity of autophagy collected from cell culture models may apply to in vivo settings as well.

The finding that autophagy could be capacity limited, especially when involving NP, could potentially bear important health consequences. Under the common condition of chronic low-level exposure to ambient air pollution UFP (and other engineered NP, particularly in the workplace), autophagic capacity may already be reached and unable to fully respond to subsequent stressors, thereby rendering the biological system more susceptible to cellular damage leading to disease due to autophagic overload. Autophagy is considered to be a cell protective mechanism [25,26,55], although it has been shown that tumor cells can use autophagy to fight for survival against injury [56]. This would also explain, at least in part, the antitumor effect of Rapamycin [40]. In addition, since we focused in this study on only one cell type to identify capacity limitation in autophagic flux, it must be noted that this phenomenon may be a unique feature of A549 cells. Further work is needed using additional cell types to be able to generalize this concept.

In summary, we have shown time-dependent activation of autophagy in response to nanoparticle exposure with and without Rapamycin. Autophagic flux was observed with unchanged steady state levels despite different exposures and combinations thereof (single agent, dual agent concurrently or sequentially), implying that autophagic activity has a maximal capacity at least in these lung adenocarcinoma cells for up to 24 hrs of exposure. These data suggest that environmental stressors may exert their harmful effects, at least in part, by exhausting or limiting available autophagic capacity, thereby making exposed lung cells more susceptible to secondary injury due to autophagic overload.

## 4. Materials and Methods

### Materials

PNP (20 nm diameter, carboxylated and impregnated with near infrared dye) was obtained from Thermo Fischer Scientific (Waltham, WA). UFP (diameter <0.18 μm) were collected from air samples in downtown Los Angeles, CA, USA per the protocol published elsewhere [57]. Transwell filters of 10.5 mm diameter (with 0.4 µm diameter pores), fetal bovine serum (FBS) and bovine serum albumin (BSA) were purchased from BD Biosciences (Franklin Lakes, NJ). A 1:1 mixture of phenol red-free Dulbecco’s modified Eagle’s medium and Ham’s F-12 medium (DME/F-12), nonessential amino acid solution (NEAA), N-(2-hydroxyethyl)-piperazine-N’-(2-ethanesulfonic acid) hemisodium salt (HEPES), dimethylsulfoxide (DMSO), L-glutamine, trypsin-ethylenediaminetetraacetic acid (EDTA) and chloroquine were all obtained from Sigma-Aldrich (St. Louis, MO). Primocin was purchased from InvivoGen (San Diego, CA). Tomato lectin, obtained from Vector Laboratories (Burlingame, CA), was labeled in-house using Dylight 405 NHS Ester labeling kit (Thermo Fischer Scientific). Premo Autophagy Sensor LC3B-GFP (catalog # P36235) and Premo Tandem Sensor RFP-GFP-LC3B (catalog # P36239) were purchased from Thermo Fischer Scientific. Rapamycin was obtained from Selleck Chemicals (Houston, TX). Autophagosome marker DAPRed was obtained from Dojindo Molecular Technologies (Washington, DC). A549 cells were purchased from American Type Culture Collection (Manassas, VA).

### Cell culture

A549 cells were plated onto Transwell filters at 100,000 cells/0.865 cm^2^ and grown in culture fluid (MDS) comprised of 10% FBS and DME/F-12 medium supplemented with 1 mM NEAA, 100 U/ml Primocin, 10 mM HEPES, 1.25 mg/ml BSA and 2 mM L-glutamine. Cells were maintained at 37°C in a humidified atmosphere of 95% air and 5% CO_2_ and fed every other day. Experiments were performed using A549 cells on culture days 4-5.

### Assessment of autophagic flux using DAPRed

In the presence or absence of chloroquine (40 μM), A549 cells were exposed to DAPRed (0.5 μM, 30 min; ex/em: 561/570-600 nm) to quantify labeled autophagosomes and autolysosomes [46,58–61]. Fluorescence intensity of DAPRed is proportional to the quantity of autophagosomes and autolysosomes at a given time point [39]. Autophagic flux at a given time point under each experimental condition (i.e., exposure to PNP, UFP and/or Rapamycin) was determined by live cell imaging as the fluorescence intensity of DAPRed measured over the entire volume of exposed single A549 cells in the presence of chloroquine (added to bathing fluids 1 hr prior to each time of measurement) minus that in the absence of chloroquine. All autophagic flux data were corrected for time point matched control autophagic flux [62]. Calculations of autophagic flux were performed as shown in Equations 1 – 2 below:

### Assessment of autophagic flux using RFP-GFP-LC3B

A549 cells were transduced by a Premo Autophagy Tandem Sensor RFP-GFP-LC3B (25 viral particles per cell) overnight. Media were replaced with fresh culture fluid for 24 hr to allow cells to recover from transduction, and on the following day A549 cells were exposed to PNP, Rapamycin or Rapamycin + PNP for up to 24 hr. Autophagic flux at a given time point under each experimental condition was determined by live cell imaging as the fluorescence intensity of LC3B-GFP (ex/em: 488/490-550 nm, signal that colocalized with LC3B-RFP (ex/em: 561/570-700 nm)) measured over the entire volume of an exposed single A549 cell in the presence or absence of chloroquine. Cells were exposed to chloroquine (40 μM) for 1 hr prior to imaging. For autophagic flux calculations, Equations 1 - 2 were used as for DAPRed (see above).

(1) $$\Phi_{control}=F_{control\left(with\;\;\;chloroquine\right)}-F_{control\left(without\;\;chloroquine\right)}$$

(2) $$\Phi_{exposure}=({\text{F}}_{exposure\left(with\;\;\;chloroquine\right)}-{\text{F}}_{exposure\left(without\;\;chloroquine\right)})-\Phi_{control}$$

**Φ: autophagic flux**

**F: DAPRed fluorescence intensity**

**exposure: PNP, UFP and/or Rapamycin**

### Colocalization of DAPRed with LC3B-GFP in live A549 cells

To further validate results with DAPRed by colocalization with LC3, A549 cells (80% confluence) were transduced with Premo Autophagy Sensor LC3B-GFP (25 viral particles per cell) overnight. After transduction, cells were provided with fresh culture fluid and allowed to recover for an additional day. Colocalization of DAPRed and LC3B-GFP (ex/em: 488/490-550 nm) was assessed by confocal microscopy and integrated over entire live single A549 cells after 24 hrs of 50 nM Rapamycin exposure. DAPRed positive and LC3B-GFP positive puncta were counted to determine colocalization of DAPRed and LC3B-GFP.

### Live cell imaging

Cells were imaged by confocal laser scanning microscopy (CLSM) as described in detail elsewhere [42]. Briefly, A549 cells on Transwell filters were mounted in a temperature-controlled chamber (Vestavia Scientific, Vestavia Hills, AL) and bathed with MDS on both sides. In xyz series, intracellular fluorescence intensity was measured stack-by-stack and integrated over the entire volume of a single A549 cell. To demarcate intracellular space at the single cell level, cell plasma membranes were labeled using Dylight 405 nm-conjugated tomato lectin. Confocal imaging was performed at 63x magnification and 1024x1024 resolution with a SP8 confocal microscope system (Leica Microsystems, Wetzlar, Germany). Gallium nitride (405 nm), argon (488 nm), diode-pumped solid state (561 nm) and helium-neon (633 nm) lasers were utilized for excitation. Image analysis was conducted using Image-J software (NIH, Bethesda, MD) and Leica LAS 3D Process and Quantify Packages (Leica Microsystems).

### Experimental design

Autophagic activity was determined in the absence and presence of 40 μM chloroquine (added to both apical and basolateral fluids 1 hr prior to each time point of fluorescence measurements) in four different experimental settings: (1) control, in which cells were exposed apically only to MDS at t = 0; (2) single agent exposure, in which cells were exposed apically to 80 μg/mL PNP, 1 μg/mL UFP or 50 nM Rapamycin at t = 0; (3) concurrent double agent exposure, in which cells were exposed apically to 80 μg/mL PNP and 50 nM Rapamycin or 1 μg/mL UFP and 50 nM Rapamycin at t = 0; and, (4) sequential (in both orders) double agent exposure, in which cells were exposed apically to a first agent (PNP, UFP or Rapamycin) at t = 0 for 5 hrs, after which cells were apically exposed to both the first agent and a second agent (Rapamycin after PNP (or UFP) and PNP (or UFP) after Rapamycin). Autophagic flux was calculated at each time point from these data as described above. The experimental designs are illustrated in the schematic diagram below:

### Data Analysis

Data are presented as mean ± standard deviation (SD) with n (total number of observations). One-way analysis of variance (ANOVA) followed by Bonferroni post-hoc procedures using Prism (version 9; GraphPad Software, San Diego, CA) was performed to determine differences among means of >2 groups. p<0.05 was considered statistically significant.

## Supplementary Material

Supplemental Material
