# Supplementary figures and images for: Kinetics of autophagic activity in nanoparticle-exposed lung adenocarcinoma (A549) cells
Source: Autophagy Rep. 2023 Mar 15;2(1):2186568. doi: 10.1080/27694127.2023.2186568 (PMC10373127; doi:10.1080/27694127.2023.2186568)

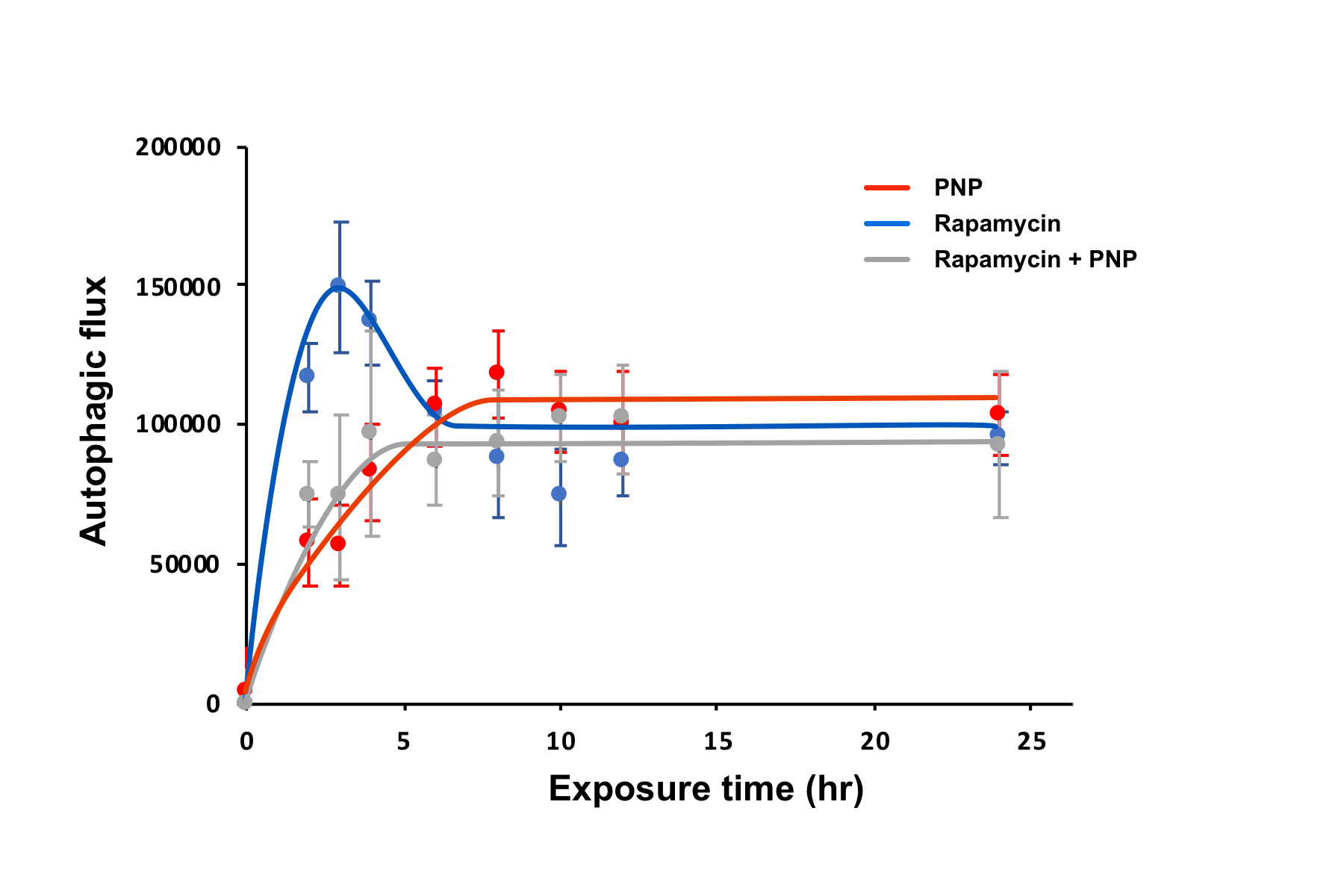

Supplement: Supplemental Material [file KAUO_A_2186568_SM0964.zip › Supplementary figure 2 (1).jpg]

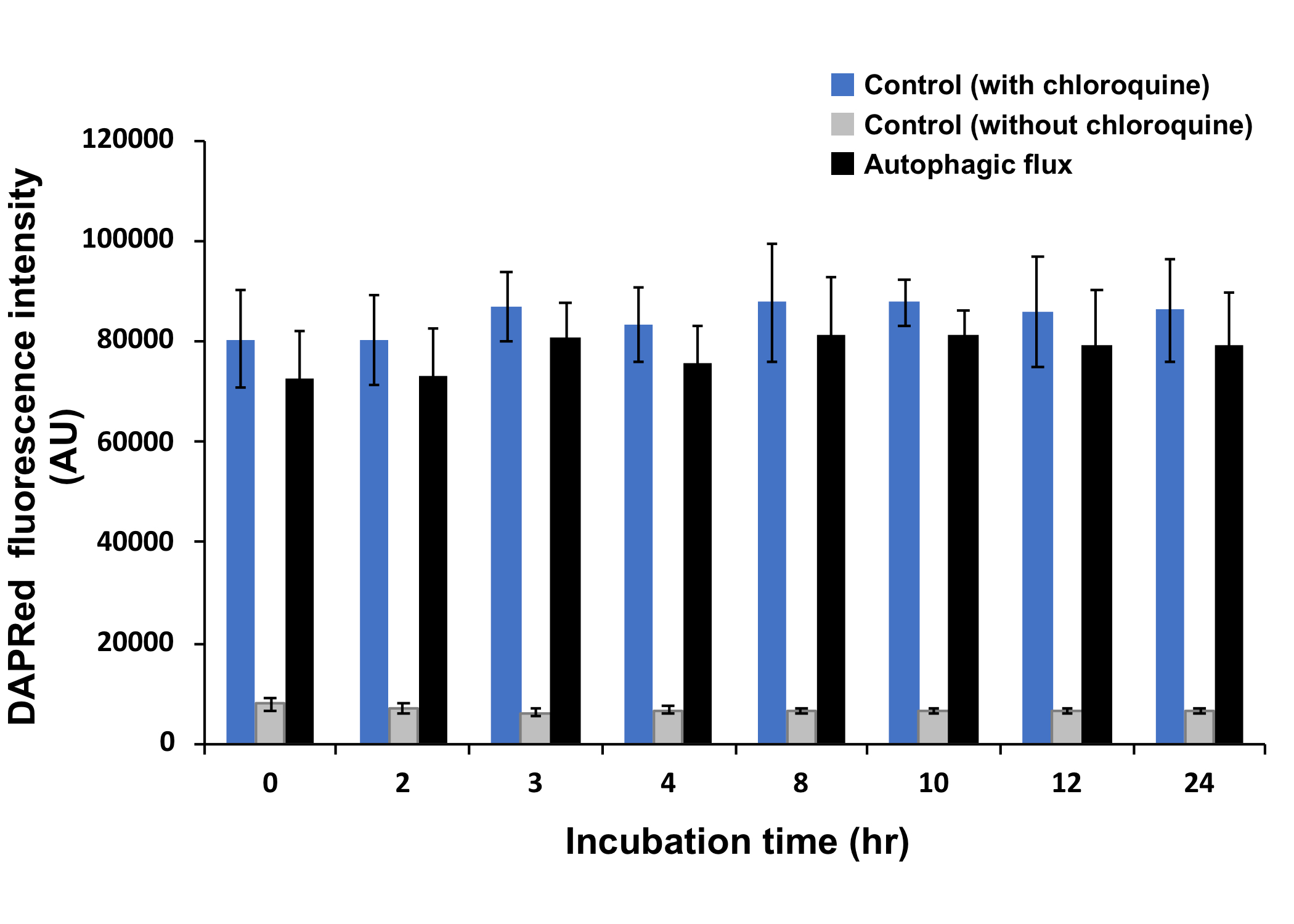

Supplement: Supplemental Material [file KAUO_A_2186568_SM0964.zip › Supplementary figure 3 (1).jpg]

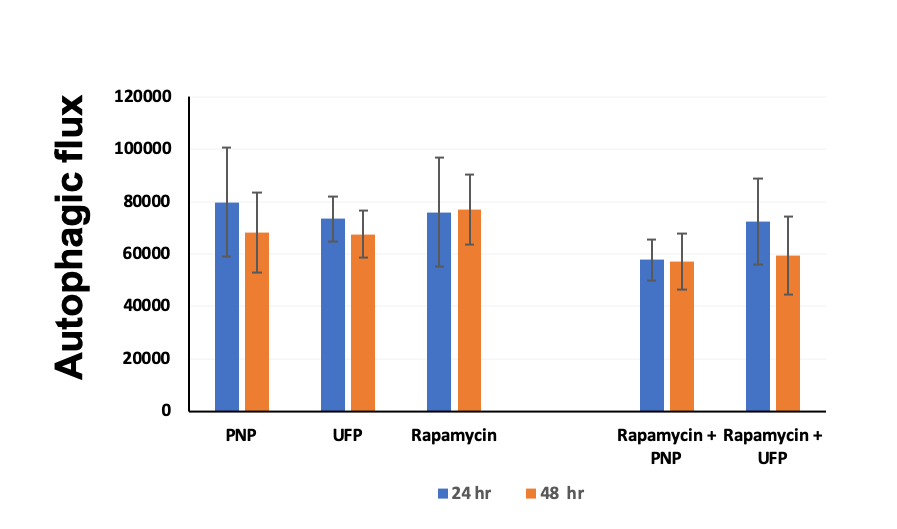

Supplement: Supplemental Material [file KAUO_A_2186568_SM0964.zip › Supplementary figure 4.png]
